# Molecularly Imprinted Microspheres in Active Compound Separation from Natural Product

**DOI:** 10.3390/molecules29174043

**Published:** 2024-08-26

**Authors:** Husna Muharram Ahadi, Firghi Muhammad Fardhan, Driyanti Rahayu, Rimadani Pratiwi, Aliya Nur Hasanah

**Affiliations:** 1 Pharmaceutical Analysis and Medicinal Chemistry Department, Faculty of Pharmacy, Universitas Padjadjaran, Bandung 45363, Indonesiarimadani.pratiwi@unpad.ac.id (R.P.); 2 Drug Development Study Center, Faculty of Pharmacy, Universitas Padjadjaran, Bandung 45363, Indonesia

**Keywords:** molecularly imprinted microspheres, active compound, natural product

## Abstract

Molecularly Imprinted Microspheres (MIMs) or Microsphere Molecularly Imprinted Polymers represent an innovative design for the selective extraction of active compounds from natural products, showcasing effectiveness and cost-efficiency. MIMs, crosslinked polymers with specific binding sites for template molecules, overcome irregularities observed in traditional Molecularly Imprinted Polymers (MIPs). Their adaptability to the shape and size of target molecules allows for the capture of compounds from complex mixtures. This review article delves into exploring the potential practical applications of MIMs, particularly in the extraction of active compounds from natural products. Additionally, it provides insights into the broader development of MIM technology for the purification of active compounds. The synthesis of MIMs encompasses various methods, including precipitation polymerization, suspension polymerization, Pickering emulsion polymerization, and Controlled/Living Radical Precipitation Polymerization. These methods enable the formation of MIPs with controlled particle sizes suitable for diverse analytical applications. Control over the template-to-monomer ratio, solvent type, reaction temperature, and polymerization time is crucial to ensure the successful synthesis of MIPs effective in isolating active compounds from natural products. MIMs have been utilized to isolate various active compounds from natural products, such as aristolochic acids from *Aristolochia manshuriensis* and flavonoids from *Rhododendron* species, among others. Based on the review, suspension polymerization deposition, which is one of the techniques used in creating MIPs, can be classified under the MIM method. This is due to its ability to produce polymers that are more homogeneous and exhibit better selectivity compared to traditional MIP techniques. Additionally, this method can achieve recovery rates ranging from 94.91% to 113.53% and purities between 86.3% and 122%. The suspension polymerization process is relatively straightforward, allowing for the effective control of viscosity and temperature. Moreover, it is cost-effective as it utilizes water as the solvent.

## 1. Introduction

Traditional practitioners have long utilized herbal plants as a source of active compounds to treat various ailments, both empirically and through generational knowledge [1]. Natural medicine has a rich history in systems such as Traditional Chinese Medicine (TCM), Traditional Korean Medicine (TKM), Ayurveda, Kampo, Unani, and Jamu, all of which heavily rely on natural products. To this day, these products remain relevant and are continually explored in modern research for new drug development [2,3,4]. The use of natural products is widespread globally, in both developed and developing countries [5]. Approximately two-thirds of drugs approved worldwide are derived from plants, with natural products playing a crucial role in the history and development of new molecular entities (NMEs) approved by the FDA [6,7,8].

Natural products, such as herbal remedies, have become prevalent in Indonesia, Ghana, and Saudi Arabia, particularly during the COVID-19 pandemic. In both Ghana and Indonesia, herbal medicine is regulated and widely utilized, with over 70% of the population relying on these products, primarily for immune system enhancement [9,10]. In a study in Saudi Arabia, approximately 46.9% of participants reported regular use of Taib, while 45.8% used it sporadically for prevention and treatment [11]. In Japan, the tradition of using natural products through Kampo is deeply ingrained, with significant integration into the healthcare system [12,13]. Meanwhile, around half of the population in the United States has tried natural remedies, as evidenced by various indicators, including herbal product sales exceeding USD 5.3 billion in 2011, reflecting a substantial market for herbal supplements. Over one-third of respondents in a study in the U.S. reported using herbal medicine, and the cultural tendency to view natural products as safer and healthier further supports this claim [14,15].

Natural products are compounds or substances produced by living organisms and found in nature, encompassing all materials generated by life, including biotic substances, biomaterials, bodily fluids, and other natural materials. These products can be classified based on their biological function, biosynthetic pathways, or sources. Natural products are typically divided into primary and secondary metabolites, with primary metabolites being crucial for the organism’s survival and secondary metabolites providing evolutionary advantages through their effects on other organisms [16,17,18].

Natural products have played a crucial role in the development of modern pharmaceuticals, particularly in the treatment of cancer and infections [18]. For example, paclitaxel, derived from the Pacific yew tree, is used in the treatment of various cancers, while artemisinin from Artemisia annua is vital in malaria therapy [19,20,21]. Captopril, isolated from Brazilian pit viper venom, serves as an antihypertensive medication, whereas simvastatin, lovastatin, pravastatin, and atorvastatin, derived from fungi, are effective in lowering cholesterol levels. Fingolimod, also from fungi, is used to treat multiple sclerosis, and aspirin, sourced from willow bark, functions as an anti-inflammatory and analgesic [10,22,23]. Natural products offer a range of pharmacological benefits, including antiviral, antibacterial, and anti-inflammatory effects, making them valuable sources for drug development. Moreover, natural products often come from renewable sources, rendering them more sustainable compared to synthetic drugs, which is important in the context of environmental conservation and circular economy [24]. Natural products also make significant contributions across various industries such as agriculture, food, cosmetics, and pharmaceuticals, providing environmentally friendly solutions that support sustainable practices and mitigate the impact of synthetic chemicals [25].

Natural products and synthetic drugs each have their advantages and disadvantages. Natural products, which originate from nature, are generally considered to have fewer side effects and lower costs. However, if not used carefully, some natural products can be highly toxic. In contrast, synthetic drugs, produced in a laboratory, are often more potent and concentrated, but they may pose more severe side effects and overdose risks. The choice between the two usually depends on individual therapeutic needs and patient conditions [26,27,28]. Although natural and synthetic drugs can share similarities in molecular structure and complexity, natural products often have limitations in the number of active ingredients and require lengthy extraction processes [23,29]. This process is crucial for obtaining pure bioactive components and for further studies on structure, activity, and toxicity. A primary challenge in extraction is the complexity of plants and variability in components, which often complicates the separation of active ingredients. Additionally, interference from endogenous matrices in the analysis of natural compounds highlights the need for simpler and faster methods to isolate active components from plants [10,23,29,30,31]. Therefore, alternative methods are needed to address these limitations.

Molecularly Imprinted Polymer technology represents an effective alternative for compound separation due to its high selectivity, and it is currently being developed as a more efficient isolation method [32]. MIP does not require additional steps beyond the extraction process to isolate a target compound [33]. MIP is a polymer created with a template that has high affinity and selectivity for the analyte molecules compared to conventional isolation methods such as liquid–liquid extraction, Soxhlet extraction, and others [34]. Research on MIPs in plant sciences has advanced significantly, with applications in isolating and identifying active compounds from plant metabolites. This technique has successfully isolated active compounds with yields ranging from 80 to 95% and accuracies reaching 92.0–100.5% [35,36,37,38,39,40]. However, conventional MIP production, typically through bulk polymerization, often results in irregularly shaped particles with low affinity, permeability, and suboptimal performance [41,42].

Molecularly Imprinted Polymers produced in the form of microspheres, or Molecularly Imprinted Microspheres (MIMs), exhibit enhanced affinity and selectivity compared to those synthesized through traditional polymerization methods. This difference can significantly influence the performance of MIPs when compared to their microsphere counterparts [43,44,45]. MIMs are highly effective for the extraction and purification of plant compounds and bio-pharmaceutical analysis. In this context, we aim to explore synthesis methods for producing MIMs in microsphere form and to identify critical variables for isolating active compounds from natural products [44,46,47,48,49]. However, to date, there has been no comprehensive review addressing the synthesis of MIMs for the separation of active compounds from natural products, despite the significant role MIMs play in enhancing the efficiency of isolating active substances from natural sources. Therefore, this review seeks to fill this information gap and provide in-depth insights into the applications of this technology and its potential for further development in the pharmaceutical sector and related fields.

## 2. Synthesis and Application of Molecularly Imprinted Microspheres (MIMs) from Natural Product

**Figure 1:**
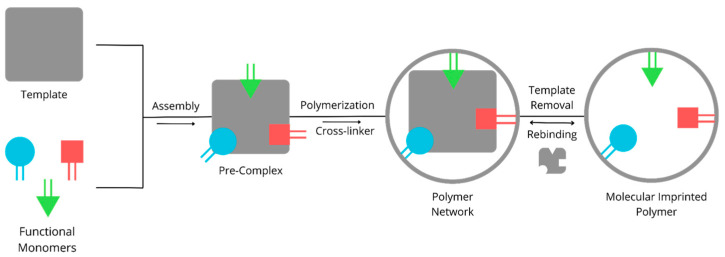
Principle of MIP.

The Molecular Imprinting Technique (MIT) is a method used to create artificial recognition sites within a polymer matrix that can specifically and selectively bind to an analyte (template molecule) in terms of the size, shape, and spatial arrangement of functional groups [50,51,52]. The MIT is often likened to the simulation of interactions between enzymes and their substrates, antibodies and antigens, as well as receptors and intracellular hormones. It is analogous to a “lock and key” model, where the MIT can selectively bind to a specific template molecule during production, forming small molecules that are resistant to various environmental conditions [43,53,54,55,56]. This concept was first introduced by Mosbach in 1993, characterized by three key attributes: structure–effect determination, specific identification, and extensive practicality [6,57]. The components involved in the fabrication of MIPs (Molecularly Imprinted Polymers) include the template molecule, functional monomers, crosslinkers, porogen, and initiators [58,59,60]. The process of MIP production involves the co-polymerization of functional monomers and crosslinking agents with a pre-existing template molecule. The template molecule is then removed, leaving behind a specific 3D cavity that matches the template molecule within the MIP [1,50,61,62]. A schematic of the MIP process is shown in Figure 1.

Conversely, non-imprinted polymers (NIPs) are used for non-specific recognition or as a negative control for MIPs to compare their performance [1,60,61]. MIPs can be produced through two main methods: covalent and non-covalent. In the covalent method, the template molecule is covalently bonded with the monomer before polymerization. Once polymerization is complete, the template is removed, leaving behind cavities that are highly specific to the template’s shape. Although this method produces highly selective and homogeneous binding sites, it is quite complex, and template removal can be challenging. On the other hand, non-covalent imprinting involves the formation of non-covalent bonds, such as electrostatic interactions, van der Waals forces, hydrogen bonds, or π-interactions, between the template and the monomer. This method is more flexible as it relies on weaker interactions, providing greater structural control, simplifying template removal, and offering dynamic properties like self-healing capability. Additionally, the semi-covalent imprinting method offers a balance between these two approaches, aiming to enhance selectivity while maintaining simplicity [63,64,65,66,67].

MIP technologies are considered highly promising due to their numerous advantages, such as exceptional thermal and chemical stability, even under extreme pH and temperature conditions. They are relatively straightforward to produce, offer high selectivity and affinity, are resistant to organic solvents, and are responsive to acidic or basic reactions. Furthermore, MIPs have broad applications across various fields, including forensics, sensor systems, chemical detection in food, plant component extraction, and the molecular recognition of biological and chemical entities [55,68,69,70,71,72,73]. Economically, MIPs are more cost-effective than other biological receptors and do not require special handling during storage. They only require a pure sample of the target molecule, enabling easier synthesis of highly specific materials at a lower cost. MIPs also hold significant potential for the extraction, isolation, and identification of bioactive components in plants [1,43,61,74].

**Figure 2:**
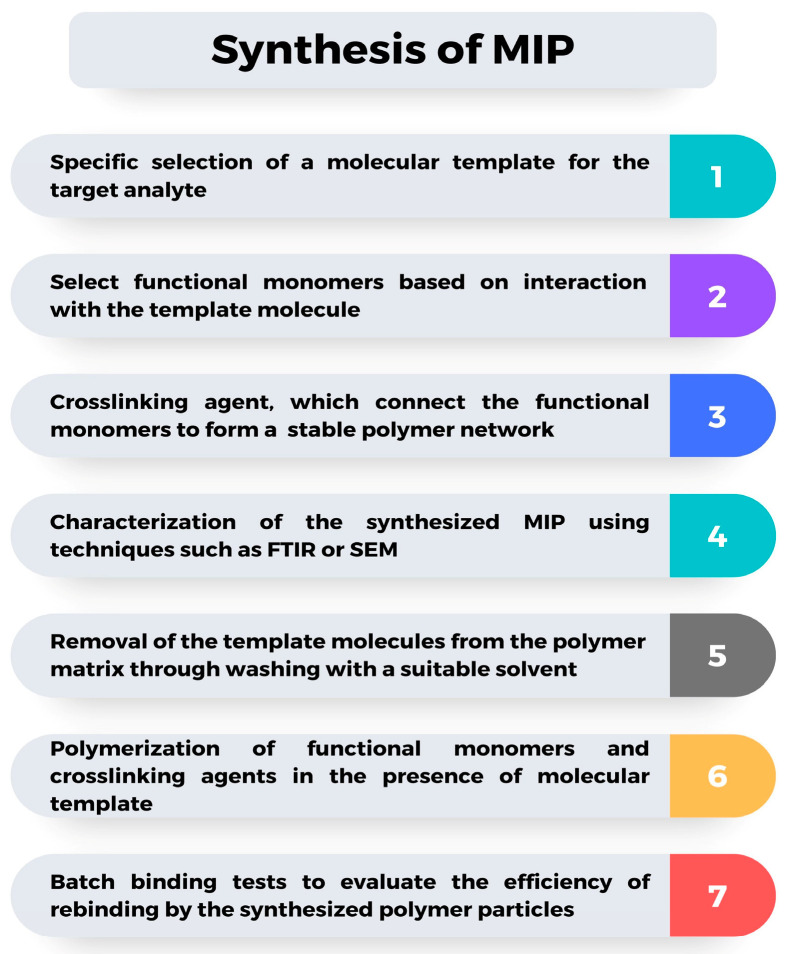
General flow of MIP synthesis.

The design of the MIP synthesis process is crucial for producing a final product with strong analytical performance. Post-imprinting modifications on MIPs allow for further adjustments to the polymer structure and enhance the affinity between the MIP and the target molecule [75,76]. One of the most critical factors is the selection of reagents, particularly the functional monomer and crosslinking agent, as these influence the affinity and specificity of the final product. The functional monomer and crosslinking agent play a vital role in determining how well the MIP can mimic the function of biological receptors. For example, functional groups such as amino or carboxyl groups on the template are essential for forming complexes with the monomer during polymerization. Additionally, the chemical stability of the template molecule is crucial to ensure that the polymerization process is not disrupted. The appropriate functional monomer will selectively interact with the template molecule, enhancing the binding capacity and selectivity of the MIP, while the crosslinking agent ensures the stability of the polymer structure after the template is removed, affecting the mechanical strength and thermal stability of the MIP [77,78,79]. Moreover, organic solvents exhibit superior specific binding capabilities compared to aqueous solvents [44]. To ensure the optimal selection of reagents, computational analysis methods such as Density Functional Theory (DFT), Molecular Docking, and Molecular Dynamics are increasingly used to predict interactions between the monomer and template, aiding in the selection of the optimal combination for high selectivity. Computational studies can predict non-covalent interactions, optimize synthesis conditions, and forecast the physical properties of the MIP, thereby accelerating the design process and improving production efficiency [63,64,80,81,82]. The general flow of MIP synthesis is outlined in Figure 2.

MIPs can be synthesized using various methods like bulk polymerization, precipitation polymerization, suspension polymerization, emulsion polymerization, two-step swelling polymerization, electropolymerization, electrospinning, sol–gel imprinting, and phase inversion, among others [83,84,85,86,87,88,89]; however, not all of these methods can be classified as MIMs. For example, bulk polymerization, known as a conventional method for creating MIPs, requires grinding and sieving during production. This process can result in a loss of approximately 50–75% of valuable polymer from the raw material and may damage some interaction sites during grinding, leading to irregular shapes, low affinity, weak binding strength, and incomplete removal of template molecules. These issues limit the extraction efficiency and imprinting capability [46]. Additionally, conventional MIPs exhibit exothermic properties, meaning that scaling up production can lead to problems with excessive heat generation in the sample. Therefore, innovative approaches are needed to overcome these limitations. MIMs can produce smaller particles, approximately <75 μm, with more regular structures [90,91,92].

Microspheres, referring to microparticles, are spherical particles with sizes ranging from 1 to 100 μm, which can be easily separated from various media [48,93,94]. Microspheres must meet several requirements, including specific size, large surface area, diffusion capability, stability, biocompatibility, and safety. Polymer microspheres should have a minimum length of 5 nanometers and a minimum molecular weight of 10,000 Da. For instance, microspheres with a diameter of 0.1 μm have a surface area of approximately 60 square meters per gram [44]. This surface area influences the chemical reactions that can occur on the microsphere’s surface [95,96]. Therefore, Molecularly Imprinted Microspheres (MIMs) can be summarized as crosslinked polymers with uniform particle sizes ranging from 0.1 to 100 μm, featuring specific binding sites for printed molecules, thus exhibiting higher affinity and selectivity for analyte molecules that act as template molecules [92,93,97,98]. MIMs find various applications, such as in competitive ligand binding assays, solid-phase microextraction (SPME), microsphere sensors, and capillary electrochromatography [92,95]. MIMs are also utilized in other applications, such as sustained drug release. MIMs are likely to have higher specificity for template molecules and greater adsorption capacity compared to Molecularly Imprinted Polymers (MIPs) [99]. Microspheres with sizes between 1.5 and 3 μm are considered suitable for a range of analytical applications [100].

MIMs are generally synthesized using various techniques such as controlled radical precipitation polymerization, precipitation polymerization, Pickering emulsion polymerization, and suspension polymerization. A comparison between MIPs and MIMs is provided in Table 1.

Polymer microsphere synthesis can be achieved through various methods, each providing control over specific characteristics and applications. There are two primary approaches for synthesizing microspheres: the first utilizes pre-existing polymer chains, and the second forms microspheres from a monomer solution through a polymerization process. These methods allow for the production of microspheres from both synthetic and naturally derived polymers, providing flexibility in material selection [49,104].

The synthesis of microspheres from natural polymer chains (the first approach) does not involve radical polymerization processes, such as those used for poly(lactic acid), poly(glycolic acid), or poly(ε-caprolactone). Instead, this method relies on solvent evaporation. Typically, the polymer is dissolved in a volatile solvent (aqueous phase) and then carefully dropped into a non-miscible liquid (oil phase). Mixing these two phases results in the formation of polymer–water droplets in the oil (continuous phase). Intermolecular forces then stabilize the microspheres, leading to the denaturation of the aggregates and making the polymer insoluble in the aqueous phase. Stabilizers are crucial for ensuring the formation and maintenance of spheroid shapes. As the solvent evaporates, solid polymer microspheres remain dispersed. These microspheres can be precipitated and washed to remove stabilizers. Crosslinking agents may be added to bond the polymers together, ensuring the mechanical stability of the microspheres. Examples of natural polymers include cellulose, chitosan, DNA, starch, silk, wool, chitin, natural rubber, and proteins such as collagen and albumin [48,105].

The main advantage of solvent evaporation for microsphere synthesis is its relative simplicity; almost any soluble polymer can be used, and high yields can be achieved. However, controlling the size of the spheroid particles between batches can be challenging. Additionally, solvents and surfactants are often toxic and must be removed before in vivo applications. The washing steps are time-consuming and generate large volumes of waste. Furthermore, drug-loaded microspheres may lose some payload during these washing steps. Spray drying offers an alternative to solvent evaporation and is straightforward to scale up. However, it produces microspheres with high polydispersity, and the relatively high temperatures required for drying can severely damage more sensitive bioactive compounds [48]. In addition, MIMs have advantages and disadvantages, which we list in Table 2.

The second approach involves forming microspheres from a monomer solution through radical polymerization. This approach relies on a mixture of monomers and polymerization initiators shaped into spheroids within an insoluble phase. We have not found any molecular imprinting (MIM) synthesis that separates active compounds from natural products using the first approach (naturally formed polymers). Therefore, we will focus on the second approach, which involves producing MIMs from a monomer solution with a polymerization initiator, forming spheroids in an immiscible phase.

## 3. Radical Polymerization-Based Synthesis of Molecularly Imprinted Microspheres

Various methods can be employed, including Controlled/Living Radical Precipitation Polymerization, suspension polymerization, precipitation polymerization, and Pickering emulsion polymerization. All of these methods fall under the second approach for forming microspheres from a monomer solution [48]. Each method will be discussed in the following sections.

### 3.1. Controlled/Living Radical Precipitation Polymerization (CRPP)

The polymerization technique that combines the advantages of controlled radical polymerization and precipitation polymerization enables the creation of highly advanced functional polymers with precisely controlled structures and properties. Controlled/Living Radical Precipitation Polymerization has been utilized to synthesize Molecularly Imprinted Polymers (MIPs) with various characteristics, including template-binding properties responsive to diverse stimuli and a strong affinity for target analytes [110]. CRPP demonstrates outstanding performance in terms of polymerization degree and control over polymer structure, leading to enhanced homogeneity in the crosslinking structure of MIP particles [111,112,113].

Controlled or living polymerization is a process where polymerization occurs in a controlled manner. In this method, side reactions such as termination and transfer are significantly minimized, allowing the produced polymer to grow consistently with monomer conversion, resulting in the linear growth of polymer chains [99,114,115]. RPP techniques employ controlled radical polymerization methods such as Atom Transfer Radical Polymerization, Reversible Addition–Fragmentation Chain Transfer, and Iniferter-Induced ‘Living’ Radical Polymerization to regulate polymer formation. The precipitation polymerization method yields uniform polymer particles with customizable properties. However, CRPP requires monomers capable of undergoing controlled radical polymerization, which limits the choice of applicable monomers [116,117]. This process also involves crosslinking agents like ethylene glycol dimethacrylate (EGDMA) to establish a robust polymer network. Initiators like azobisisobutyronitrile (AIBN) are employed to initiate the polymerization reaction. Subsequently, solvent washing removes template molecules from the polymer matrix, leaving specific binding sites for the target analytes [118].

Controlled Radical Polymerization (CRP) techniques depend on the concept that reactive radicals can be temporarily deactivated by forming covalent terminals. This capability ensures controlled reaction rates, enabling most monomers to react and resulting in chains of similar lengths [119,120]. Although ATRP is suitable for films of specific thicknesses [113,121], RAFT excels in large-scale production as it does not require specific catalysts or environmental conditions like ATRP. The advantages of CRPP include milder reaction conditions, the ability to produce uniform and well-defined microspheres from functional monomers, and applicability to a wide range of monomers and crosslinkers. Disadvantages include limited monomer selection, scale stability issues, longer reaction times, and higher costs [122,123,124].

Based on research by Alipour et al. [125], nanoscale MIPs with a microporous structure were successfully synthesized for the selective and efficient adsorption of rosmarinic acid (RA) from *S. officinalis* plant extracts. Characterization was conducted using Fourier Transform Infrared Spectroscopy (FTIR), Scanning Electron Microscopy (SEM), and Brunauer–Emmett–Teller (BET) techniques. The analysis revealed that the MIP particles, measuring 88 nm in size, exhibited a uniform and spherical shape. The highest recovery percentage (>90%) was achieved after preparing an RA solution with a pH of 5.7, followed by elution with 3 mL of acetic acid solution and sonication for 6 min on the formed nanobeads. Selectivity analysis demonstrated that the template molecule (RA) exhibited high selectivity and affinity towards the MIP absorbent compared to other compounds such as gallic acid, caffeic acid, and pyrocatechol.

In the MIP made by Pardeshi et al., [126], the recovery was high. Through application testing by extracting gallic acid from *E. officinalis* plant, a recovery value of 96–98% was obtained with a selectivity level of 75–83.4%. These results show that MIPs can extract a compound from a complex sample and successfully achieve a high level of recovery and selectivity. Examples and summaries of results from MIPs using the CRPP method can be seen in Table 3.

#### 3.1.1. Atom Transfer Radical Precipitation Polymerization (ATRPP) 

Atom Transfer Radical Precipitation Polymerization combines the Atom Transfer Radical Polymerization (ATRP) system with the PP (precipitation polymerization) procedure, replacing conventional initiators (such as AIBN) with ATRP initiators. After activation under appropriate reaction conditions, all chains initiate rapidly and grow simultaneously, forming soluble branched oligomers at the onset of polymerization. Controlled characteristics of ATRPP play a crucial role in uniform particle growth [127,128]. This technique is continually being developed to create high-quality polymer microspheres. The first applied the ATRP mechanism to PP, creating “living” polymer microspheres with an average diameter ranging from 0.73 to 3.25 μm and a low polydispersity index. Others combined reverse ATRP with PP, obtaining “living” polymer microspheres via two PP steps, where the first step was conventional PP (for nucleation) and the second step was reverse ATRP (for particle growth) [129,130,131]. However, a novel ATRP-based methodology, ARGET ATRPP, was initially reported, producing high-quality products (in the size range of 1–3 μm) which were directly usable in grafting experiments without requiring initiator installation [131,132].

ATRP employs transition metal catalysts to form carbon–carbon bonds, with atom transfer pivotal in ensuring consistent polymer chain growth during the reaction. Within ATRP, a persistent radical exists, represented by the oxidized metal catalyst (“deactivator”) and the reduced metal catalyst (“activator”). This process can take place in various solvents, including toluene, 1,4-dioxane, xylene, anisole, DMF, DMSO, water, methanol, acetonitrile, or even the monomer itself [100,133,134]. 

There are two types of ATRP processes: normal ATRP and reverse ATRP. In normal ATRP, alkyl halides serve as initiators and transition metal complexes in a lower oxidation state (such as Cu(I)/ligand) as catalysts, directly creating the initiation radical. On the other hand, reverse ATRP uses conventional radical initiators (e.g., AIBN) and transition metal complexes in a higher oxidation state (e.g., Cu(II)/ligand) as species and initiation catalysts, respectively. Initially, radicals from the conventional initiator are neutralized by transition metal complexes with a higher oxidation state, resulting in alkyl halides and transition metal complexes with a lower oxidation state, which then kick-start controlled polymerization, similar to normal ATRP. In both systems, the equilibrium between inactive species (like alkyl halides) and active radicals rapidly forms after the polymerization commences. However, the presence of transition metal complexes in the ATRP system poses challenges in purifying the product and protecting acidic monomers before polymerization [135,136,137].

**Figure 3:**
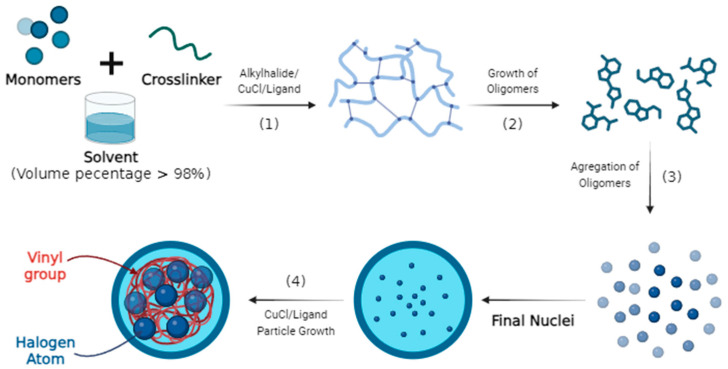
Illustration of the ATRPP mechanism.

ATRPP includes the typical steps of particle nucleation and growth. It starts from a homogeneous mixed solution of divinyl crosslinker, mono vinyl functional monomer (functional monomer with only one vinyl group), initiator, catalyst (copper halide and ligand), and a large amount of solvent. When ATRPP is activated under appropriate reaction conditions, all chains are rapidly initiated and grow simultaneously, resulting in soluble branched oligomers at the onset of polymerization. As these branched oligomer chains grow beyond their solubility limit in the reaction medium, they precipitate out of the continuous medium, and their subsequent aggregation eventually leads to the formation of a particle core (with ATRP initiator groups immobilized on the surface), which then increases in size according to the polymerization process [129,138]. The illustration mechanism of ATRPP can be seen in Figure 3.

The advantages of ATRPP are the extensive utilization of monomers, initiators, and catalysts; the resulting MIMs typically feature end-capping through reactive halogen groups; and the cost is relatively low. The disadvantage of ATRPP is that it may has restricted practicality as it involves substantial quantities of acidic functional monomers or templates, which could potentially deactivate the metal catalyst [129]. We have not yet found the application of MIMs in extracting natural compounds using this method. The probable reason as to why it is so difficult to find the application of ATRPP in natural product extraction is the difficulty and expense of removing and recycling the catalyst from the polymer after polymerization. These limitations arise from the strong bonding of the polar tip to the metal, high resistance to subsequent monomer insertion, and rearrangements that affect regioselectivity and stereoselectivity [139,140,141,142,143].

#### 3.1.2. Iniferter-Induced “Living” Radical Precipitation Polymerization (ILRPP)

ILRPP (Iniferter-Induced ‘Living’ Radical Precipitation Polymerization) incorporates a ‘living’ radical polymerization mechanism into precipitation polymerization by replacing the traditional initiator (e.g., AIBN) with an iniferter (molecules that could act as initiator, transfer agent, and termination agent, e.g., benzyl dithiocarbamate or BDC). This versatile method allows for the polymerization of a wide range of monomers under mild conditions, such as UV light irradiation at room temperature with a photoiniferter. Like traditional precipitation polymerization and ATRPP, ILRPP involves particle nucleation and growth stages. It begins with a mixture of crosslinker, functional monomer (if applicable), iniferter, and solvent. When activated under suitable conditions, all polymer chains initiate and grow simultaneously, forming soluble branched oligomers initially. As these chains exceed their solubility limit, they precipitate and aggregate to create particle nuclei with surface-bound iniferter groups, followed by a size increase during polymerization. According to the iniferter-induced ‘living’ radical polymerization mechanism, iniferters in ILRPP transform into macroiniferters early in the process. Due to the relatively low reactivity of the dithiocarbamyl radical, newly formed polymer chains in the reaction medium are minimal during particle growth. Thus, ILRPP particles primarily grow by capturing monomers directly from the reaction solution through surface-initiated ‘living’ polymerization. This ‘living’ characteristic results in uniform particle growth, precise control over particle sizes, and uniformly crosslinked polymer networks. Importantly, all resulting polymer microspheres from ILRPP feature active iniferter groups on their surfaces, enabling further surface functionalization [141,144,145,146].

According to the iniferter-induced “living” radical polymerization mechanism, all iniferters in the ILRPP system will be rapidly converted into macroiniferters immediately after the initiation of polymerization. This, combined with the relatively less reactive properties of the dithiocarbamyl radical (which can be considered non-reactive compared to carbon radicals, especially at room temperature), suggests that the amount of chain is significant. New polymers are formed in the reaction medium during the molecular growth phase, so they are insignificant. Therefore, the polymer particles in the ILRPP system will also grow mainly by direct capture of monomers from the reaction solution through “living” polymerization initiated on the surface [141,146]. 

The “living” properties of ILRPP will result in uniform particle growth, suitable particle size control, and a relatively uniform crosslinked polymer network. In particular, all polymer microspheres obtained by ILRPP must contain active depletion groups on their surface, thus allowing their subsequent surface functionalization. Advantages of ILRPP are that it is suitable for various molecular imprinting systems, and resulting MIMs typically have end-capping involving iniferter groups. The disadvantage of ILRPP is that it is less controlled compared to ATRPP and RAFTPP [146].

We have not yet found the application of MIMs in extracting natural compounds using this method. The probable reason as to why it is so difficult to find the application of ILRPP in the extraction of natural product is that ILRPP shows less control compared to other methods like Atom Transfer Radical Precipitation Polymerization (ATRPP) and Reversible Addition–Fragmentation Chain Transfer (RAFT) Precipitation Polymerization. This reduced control can lead to variations in the properties of the resulting polymer particles, potentially affecting their performance in specific applications [127,146].

#### 3.1.3. Reversible Addition–Fragmentation Chain Transfer Precipitation Polymerization (RAFTPP)

RAFT polymerization is a controlled radical polymerization technique renowned for its exceptional control over polymer structures, versatility with different monomers, and mild reaction conditions. The RAFTPP technique synergistically integrates RAFT polymerization with precipitation polymerization, allowing for controlled and customizable polymerization processes that produce intricate polymeric structures with tailored properties and functionalities [147,148]. RAFT polymerization begins similarly to conventional radical polymerization, where the cleavage of a radical initiator generates active free radicals. These radicals initiate polymerization, leading to an equilibrium between growing polymer chains and dormant RAFT compounds. This equilibrium results in well-defined polymers with specific end groups [144,149,150]. 

Compared to other controlled radical polymerization methods like ATRP (Atom Transfer Radical Polymerization) and NMP (Nitroxide Mediated Polymerization), RAFT polymerization is advantageous due to its ability to synthesize well-defined polymers from a broader range of monomers under milder conditions. It can be applied in various polymerization modes, including solution, emulsion, and suspension polymerizations. RAFTPP incorporates the RAFT mechanism into traditional precipitation polymerization by adding a suitable chain-transfer agent (or RAFT agent). This “living” polymerization approach produces microspheres with surface-immobilized dithioester groups and is promising for creating advanced functional polymer materials due to RAFT’s versatility in controlling polymer structures [149,151,152].

RAFTPP is accomplished by integrating the RAFT polymerization mechanism into conventional precipitation polymerization systems using an appropriate chain-transfer agent. This approach converts traditional systems into RAFTPP systems, where the “activity” of RAFT polymerization endows RAFTPP with dynamic properties, resulting in “active” polymer microspheres with dithioester groups on their surfaces. RAFTPP’s versatility makes it highly promising for developing well-defined advanced functional polymer materials [144].

The advantage of RAFTPP is that there is an extensive range of applicable monomers, encompassing nearly all types. The resulting MIMs are typically capped with dithioester groups; it is also particularly effective for crafting high-molecular-weight polymers. The disadvantages of RAFTPP are that the inclusion of dithioester groups imparts color to the resulting MIMs and may introduce odors, especially for low-molar-mass species that might necessitate radical chemistry for removal and displacement; and, additionally, the continuous generation of new short chains occurs, terminating more rapidly than the longer chains [144].

MIMs synthesized by Xiao et al. [148] used acrylic acid as the monomer, EGDMA as the crosslinker, AIBN as the initiator, and aristolochic acid I as the template. The binding sites on the MIMs, described by the Scatchard isotherm model, acted independently. Recycling experiments showed that the MIMs could be reused for the selective adsorption and separation of aristolochic acid I up to six times. The resulting MIM demonstrated a high recovery rate of 91.5%. Details of the conditions and results for MIMs synthesized using RAFTPP are summarized in Table 4.

### 3.2. Suspension Polymerization

Suspension polymerization occurs in two immiscible phases: the continuous phase and the dispersed phase. The dispersed phase contains functional monomers, initiators, porogens, and template molecules, while the continuous phase includes the crosslinker. Polyvinyl alcohol is often added to the continuous phase as a suspending agent to enhance stability [88,153]. In suspension polymerization, polymer particles form by dispersing monomers into a liquid medium with tiny droplets, followed by the addition of crosslinking agents and initiators to start the polymerization reactions. This creates conditions where fragmented monomer droplets can either reassemble or remain dispersed in the continuous fluid or turbulent flow during polymerization [109,154]. The tiny droplets act like small bulk polymerization units, resulting in more uniform particle shapes and sizes [88].

To synthesize the polymer, 1.0 mmol of p-HB, 4.0 mmol of 4-VP, and 10 mL of acetonitrile were combined and refrigerated at 4 °C for 12 h to create a preassembly solution. This solution was then dispersed into droplets within a 1.5 wt% aqueous poly(vinyl alcohol) solution containing sodium dodecyl sulfate. N2 gas was purged through the mixture for 5 min. Subsequently, 20 mmol of crosslinker (EGDMA) and a small quantity of initiator (AIBN) were added. After another 10 min N2 purge, the sealed mixture was sonicated at 0 °C for 30 min to disperse the polymerizable suspension into smaller droplets. Polymerization was initiated thermally in a water bath at 60 °C and continued for 24 h. The resulting MIP was collected and subjected to multiple sonication cycles in a methanol and acetic acid mixture to remove the template and any remaining monomer. Finally, the polymer was separated by centrifugation and dried overnight under vacuum at 50 °C. For comparison, a non-imprinted polymer (NIP) was prepared using the same procedure but without the inclusion of p-HB [154,155].

**Figure 4:**
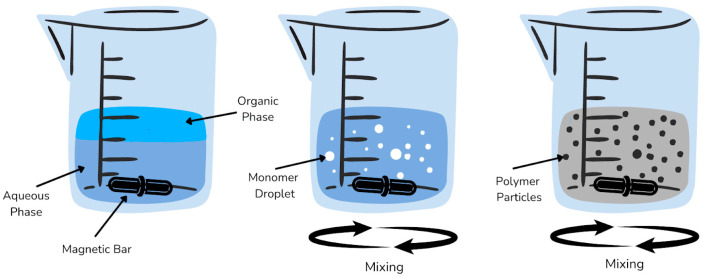
Process of suspension polymerization.

Song et al. [145] reported hydrophilic MIPs synthesized via suspension polymerization. Glycidyl methacrylate (GMA) was used to modulate the polymer’s hydrophilicity, with polyvinyl alcohol serving as the suspending agent. After polymerization, epoxy bonds were successfully formed on the surface of the polymer nanospheres. Various functional monomers were evaluated, including MAA, acrylonitrile, acrylic acid, n-butyl acrylate, n-butyl methacrylate, and methyl methacrylate. MAA proved to be an effective functional monomer due to its excellent adsorption capability onto MIPs. However, control over ring-opening reactions is necessary post-polymerization, as hydroxyl groups formed from these reactions can participate in pre-polymerization via hydrogen bonding, which may reduce MIP adsorption capacity. Further studies demonstrated that the developed MIPs exhibited outstanding performance in detecting aflatoxin B1 (AFB1) in soy sauce, with a recovery rate of 96%. The method showed a linear range of 10–1000 ng mL^−1^, with a linear coefficient of 0.9994 and a detection limit of 0.05 ng mL^−1^. Dispersants are necessary in suspension polymerization, but they are challenging to remove completely from the polymerization product, which can affect its properties. The process of suspension polymerization can be seen in Figure 4.

In suspension polymerization, monomer droplets are dispersed in water and act as separate microreactors. The polymerization is initiated with an organic initiator and proceeds as a miniature bulk polymerization reaction. As monomers are converted into polymers, the droplets transform into sticky, viscous monomer/polymer particles, gradually becoming spherical solid polymer particles. Vigorous mixing and spatial stabilization are required. The polymer particles produced are several hundred micrometers in size and settle once stirring is stopped. Suspension polymerization is particularly useful for making polymers from reactive monomers via free radical polymerization. Continuous stirring facilitates mixing and promotes heat transfer. The viscosity of the suspension remains relatively constant during monomer conversion. Polymer products must be separated from water before use. The volume fraction of the dispersed phase typically ranges from 25% to 50% [155,156].

Based on research conducted by Kang et al. [157], it was demonstrated that MIMs can effectively extract and enrich oxymatrine and matrine from deep eutectic solvent (DES) extracts. MIMs were synthesized using acrylamide as a monomer, EGDMA as a crosslinker, AIBN as an initiator, and oxymatrine as the template. The DES-based extraction followed by MIM secondary enrichment proved to be an effective method for selectively purifying oxymatrine and matrine from complex samples. Static and dynamic adsorption experiments showed an adsorption capacity for oxymatrine of 110.8 mg/g, indicating strong performance. The MIMs also demonstrated a satisfactory recovery rate, ranging from 80.21% to 89.15% for oxymatrine.

MIMs synthesized by Liu et al. [158] used MMA and 4-VBPE as monomers, EGDMA as a crosslinker, AIBN as an initiator, and ginsenoside Rb1 as a template. The resulting MIMs exhibited strong surface hydrophilicity. In aqueous media, the adsorption capacity for the template reached 81.45 μmol/g, and the elution recovery was high at 92.39%.

Ma et al. [159] synthesized MIMs using farrerol as a template, 4-vinylpyridine (4-VP) as a monomer, and EGDMA as a crosslinker. The resulting MIMs had an irregular spherical shape with wrinkled protrusions. They exhibited a pore size of 150.34 m^2^/g and a porosity of 71.77%. These MIMs demonstrated good affinity for farrerol, taxifolin, kaempferol, and hyperin compared to matrine, with extraction results showing farrerol at 80.12–105.46%, taxifolin at 82.02–95.41%, kaempferol at 75.08–89.05%, and hyperin at 64.04–78.03%.

Ma et al. [160] also synthesized MIMs using dihydroquercetin as a template, 4-VP as a monomer, and EGDMA as a crosslinker. These MIMs had a size of 250 nm and a general spherical shape with rough and smooth surfaces, including holes and a clear core–shell structure. The MIMs showed a pore size of 150.34 m^2^/g and a porosity of 71.77%. The extraction results for dihydroquercetin were 74.64–101.80%, with a maximum adsorption capacity of 77.72 mg/g.

Xie et al. [40] synthesized MIMs using protocatechuic acid (PA) as a template, acrylamide as a monomer, and EGDMA as a crosslinker. The MIMs had a size of 700–900 nm and a uniform spherical shape. They demonstrated high specificity and selectivity towards PA, with extraction results showing a recovery of 86.3–102.2%. Examples and summaries of results from MIMs using these methods are summarized in Table 5.

### 3.3. Precipitation Polymerization

Precipitation polymerization is a widely used technique in producing MIMs. This method allows for the generation of uniform MIMs when conducted under highly dilute conditions [84,161,162]. Generally, the polymerization process is similar to bulk polymerization methods but requires a significantly larger amount of solvent compared to conventional approaches, typically less than 5% of the total monomer volume. Additionally, the precipitation process sometimes occurs only in specific areas. Precipitation polymerization offers several advantages, such as the growth of individual polymer chains into microspheres, the elimination of the need for porogenic agents in the reaction mixture, and a procedure that is simpler and less time-consuming compared to conventional methods. This technique results in the formation of microspheres with an average particle diameter in the range of 0.2–0.3 μm [44,163,164].

The underlying mechanism of precipitation polymerization involves initiation in a homogeneous solution containing a monomer, crosslinker, initiator, and solvent. In conventional precipitation polymerization, the polymer formation process begins with the creation of nuclei and oligomers. These oligomers gradually grow and start to crosslink. Instead of merging, polymer chains individually expand by incorporating oligomers and newly generated monomers from the solvent. The chain length continues to increase until surpassing the solubility threshold, ultimately leading to precipitation from the solution in the form of uniform spherical structures [44,164,165].

As previously reported, conventional MIPs typically exhibit irregular shapes and varying sizes, whereas MIMs yield spherical microgels with narrow size distributions. The formation of MIMs depends on various factors. For instance, it is crucial to adjust the growing polymer solubility parameters when aiming to produce larger particles while controlling polymer morphology. Additionally, monomer concentration, solvent mixture composition, and agitation method all contribute to the development of MIMs [92,166].

MIMs synthesized by Di et al. [167] were made using acrylamide as the monomer, TRIM as the crosslinker, AIBN as the initiator, and proanthocyanidin as the template. Application tests of these MIMs for extracting proanthocyanidin from Camellia seed shells in suitable conditions resulted in recoveries of 10.34 ± 2.11% and 85.24 ± 3.05%. This result is considered good due to its high recovery rate.

MIMs synthesized by Alipour et al. [125] had uniform, spherical particles with an average size of around 80 nm and a narrow size distribution. These MIMs were synthesized using MAA as the monomer, EGDMA as the crosslinker, AIBN as the initiator, and rosmarinic acid as the template. In a selective study, MIMs achieved an 88% recovery percentage in a mixed solution (containing rosmarinic acid, gallic acid, caffeic acid, and pyrocatechol), which 4% less compared to MIMs in a rosmarinic acid solution (which had 92.4% recovery). In the application of extracting rosmarinic acid from *Salvia officinalis*, MIMs achieved 77.80% recovery, indicating high selectivity and acceptable recovery.

MIMs synthesized by Tabaraki and Sadeghinejad [168] used rutin as the template, MAA as the monomer, and EGDMA as the crosslinker. The resulting MIMs had a pore size of 2.736 nm with a BET area of 257.050 m^2^/g and high selectivity capabilities. The journal did not include test results for rutin extraction from green tea.

MIMs synthesized by Chen et al. [169] used rhaponticin as the template, acrylamide as the monomer, and EGDMA as the crosslinker. The resulting MIMs had diameters ranging from 5 to 27 µm, with the majority being spherical in shape. The selectivity ability was also high compared to the compounds resveratrol and kirenol. The results of rhaponticin extraction from Chinese patent medicine using these MIMs showed good recovery values of 77.82–91.00%.

MIMs synthesized by Gu et al. [170] used chlorogenic acid (CGA) as the template, MAA as the monomer, and TRIM as the crosslinker. The resulting MIMs had diameters of 15 and 50 µm with a shell thickness of 17 nm. The selectivity capability was high, with results 6 times greater than NIPs when compared to caffeic acid compounds. The chlorogenic acid extraction test from Traditional Chinese Medicine using these MIMs showed a recovery value of 78.85%. Examples and summaries of results from MIMs using this method can be seen in Table 6.

### 3.4. Pickering Emulsion Polymerization

Pickering emulsions are a distinct type of liquid mixture held together by small solid particles congregating at the interface between two immiscible fluids [171]. Compared to other types of emulsions, these possess remarkable stability and can be stored for extended periods without undergoing significant phase changes [172]. The fundamental principle of precipitation polymerization is that polymers form as chains grow in solution until reaching a certain critical mass. Upon reaching this critical mass, polymer chains precipitate or coalesce from the solution, forming particles or microgels. In the context of MIM synthesis, this principle is utilized to produce spherical MIM particles with specific properties for recognizing target molecules (templates) [173]. Various solid particles can be employed to stabilize Pickering emulsions. Nanoscale silica particles, for instance, are environmentally friendly, easily synthesized, and modifiable, making them one of the most extensively researched particle stabilizers for creating Pickering emulsions [174]. Magnetic nanoparticles, possessing magnetic properties and low toxicity, find wide applications in fabricating Pickering emulsions for biomedical purposes. Carbon nanotubes have also garnered attention as novel particle stabilizers due to their large surface area and abundant active sites, offering intriguing possibilities for Pickering emulsions [175]. Additionally, naturally derived particle stabilizers, known for their biocompatibility and biodegradability, are gaining popularity. The emergence of diverse particle stabilizers has opened up promising prospects for utilizing Pickering emulsions across various applied sciences [176,177].

MIMs can be synthesized via Pickering emulsions using various approaches without needing stabilizers or surfactants to yield monodisperse MIM beads. However, several factors need consideration, such as the initiator being hydrophilic, the monomer being hydrophobic, and the need for an emulsifying agent. In oil-in-water emulsions, the oil phase containing the monomer undergoes polymerization to create crosslinked polymer beads, wherein the molecular template is enclosed within or situated on the surface of the beads. Removing the template under appropriate conditions results in the formation of specific binding sites with sizes, shapes, and 3D structures mimicking the corresponding template. When microorganisms are employed to stabilize Pickering emulsions comprising dispersed hydrophobic monomer droplets in water, microbial cells can act as templates, forming imprinted sites on the surface once the monomer droplets are crosslinked into solid polymer beads. In many practical applications, ensuring accessibility to the imprinted binding sites is crucial for achieving rapid analysis results and facilitating the regeneration and recycling of valuable materials [106,178,179].

In oil-in-water Pickering emulsions, harnessing bacteria as surface templates has yielded surface-imprinted polymer beads through Pickering emulsion polymerization. The synthetic process involves self-assembling bacterial–chitosan complexes, the fabrication of complex-stabilized Pickering emulsions, and subsequent imprinting reactions. To tether cationic chitosan to polymer beads, chitosan is initially modified with acryloyl chloride to introduce C=C bonds before being added to the bacterial template. The self-assembly of bacterial–chitosan complexes is driven by electrostatic interactions between negatively charged bacteria and positively charged acrylamide-functionalized chitosan. In this manner, bacterial–chitosan complexes serve as particle stabilizers to stabilize oil-in-water Pickering emulsions, with bacteria acting as cellular templates to generate imprinted recognition sites on the surface of the resulting crosslinked polymer beads [180,181,182].

MIMs synthesized by Sun et al., [183] used 4-vinylpyridine (4VP) as the monomer, divinylbenzene (DVB) as the crosslinker, AIBN as the initiator, and quercetin as the template. The resulting MIMs had uniform spherical shapes with a diameter distribution of 55 µm. Tests conducted by Sun et al. indicated that the MIMs had an equilibrium adsorption capacity of 521 µg/g. However, the recovery rate of these MIMs was not mentioned in the journal. 

MIMs synthesized by Zhou et al. [184] used 17β-estradiol as the template, methacrylic acid (MAA) as the monomer, and ethylene glycol dimethacrylate (EGDMA) as the crosslinker. The resulting MIMs had a size of 18.9 µm with a spherical shape. Binding ability testing was carried out using several different solvents, with the best results being in water, showing a binding ability value of 45-63%. However, the recovery rate of these MIMs was not mentioned in the journal. Examples and summaries of results from MIMs using this method can be seen in Table 7.

## 4. Factors That Affect the Performance of Molecularly Imprinted Microspheres (MIMs)

In the context of MIMs, several factors can influence the process, such as the selection of monomers, efficient crosslinkers, the molar ratio, and appropriate solvents. Selecting an appropriate solvent is crucial as it determines particle formation. The use of an unsuitable solvent may result in failure to obtain the desired particles. Another aspect that affects the precipitation polymerization of MIMs is particle morphology, which encompasses factors like porosity, particle size, and polydispersity [185].

### 4.1. Functional Monomers

Functional monomers capable of polymerization with double bonds or distinctive functional groups can exhibit specific interactions with template molecules. Functional monomers and templates can be combined through either covalent or non-covalent bonding. The choice of functional monomer depends on the properties and features of the template molecule. For example, if the template possesses primary functional groups, using precursors with acidic groups, such as acrylic acid or methacrylic acid, is preferred to facilitate the formation of ionic interactions or hydrogen bonds. Conversely, if the template presents acidic groups, the best choice is to use weak bases, such as vinyl pyridine. Lastly, if the template can form stable complexes with specific bonds, the use of functional chelating monomers like vinylimidazole would be the optimal choice. The strength of interactions between the functional monomer and the template directly influences the affinity, selectivity, and accuracy of MIMs towards the target molecule [186,187,188].

The most widely used functional monomers in the synthesis of natural product derivatives are methacrylic acid (MAA) and its derivatives. MAA is an unsaturated monocarboxylic acid capable of forming stable complexes with various molecules through hydrogen bonding and weak electrostatic attraction. The use of MAA in isolating compounds from plants has led to the formation of new functional monomers, such as β-cyclodextrin-functionalized methacrylic acid (MAA-βCD). This new functional monomer can be employed in MIM fabrication to selectively remove specific contaminants, such as 2,4-dichlorophenol (2,4-DCP) [189].

### 4.2. Type of Crosslinker

Crosslinkers are organic compounds with multiple functional groups capable of forming covalent bonds with functional monomers and template molecules during the polymerization process. Crosslinkers play a crucial role in creating a stable three-dimensional network within MIMs and ensuring their structural integrity. The types of functional monomers and crosslinkers must have similar reactivity so that during random copolymerization, the functional groups of the monomers can be evenly distributed [41]. The bond between the functional monomer and the template should also be considered because improper crosslinker selection can disrupt the formed bonds. The type of polymerization is also a consideration because the presence or absence of free radicals in the polymerization process will affect the resulting MIMs [94]. Commonly used crosslinkers for free radical polymerization include EGDMA, divinylbenzene (DVB), and trimethylolpropane trimethacrylate (TRIM) for non-covalent bonds, and triallyl isocyanurate (TAIC), dicumyl peroxide (DCP), and bis-(1-tert-butylperoxy)-1-methylethyl)-benzene (BIPB) for covalent bonds [190,191]. Additionally, solvents must also be considered: the crosslinker used should be insoluble in the solvents employed. Commonly used crosslinkers for inorganic solvents are N,N-methylenebisacrylamide, and for organic solvents, they are DVB and EGDMA [192].

MIMs incorporating trifunctional crosslinking groups demonstrate greater rigidity, better structural organization, and more efficient binding sites compared to those with bifunctional crosslinkers. MIMs synthesized using ethylene glycol dimethacrylate (EGDMA) tend to have a looser structure than those utilizing divinylbenzene (DVB). This looser structure can accelerate molecular mass transfer and enhance the binding kinetics of MIMs [192]. The quantity of crosslinker directly impacts MIM performance. Insufficient crosslinking can lead to low crosslinking density, potentially causing cavitation within MIMs, instability in maintaining cavity structure, and reduced molecular recognition capability. Conversely, an excess of crosslinker reduces the number of recognition sites in MIMs, resulting in increased structural rigidity and diminished binding capacity. These crosslinkers are chosen based on their ability to form stable covalent bonds with functional monomers and template molecules, as well as their compatibility with polymerization methods and the desired properties of the final MIM [193].

There are a few types of crosslinkers. The first one is the lightly crosslinked polymer, which consists of microgels and styrene monomers. Lightly crosslinked polymers have a lower crosslinking density, which can result in larger particle sizes due to the reduced interconnectivity between polymer chains—lightly crosslinked polymers like polyacrylamide, polymethacrylic acid (PMA) microparticles, isotactic polypropylene films, and polyurethane. The second one is the highly crosslinked polymer, which consists of certain functional polymers such as ethylene glycol and divinylbenzene. Highly crosslinked polymers have a higher crosslinking density, which can cause surface shrinkage and smaller particle sizes. The increased divinyl benzene (DVB) could cooperate with the HD (δ) in the polymer, resulting in a more densely packed structure with smaller particle sizes [153,194,195].

### 4.3. Initiator

Initiators can influence microsphere imprinted polymers’ morphology, performance, and molecular recognition properties. A comparative study of imprinted polymer particles prepared by different polymerization methods showed that the choice of initiator can affect the morphology and performance of MIMs. This suggests that the initiator plays a role in determining the physical and chemical properties of the polymer. A new concept for the synthesis of MIMs using a functionalized initiator has been proposed. This approach replaces traditional initiators and offers a novel method for synthesizing MIMs with specific binding sites [196,197,198].

The initiator-free synthesis of MIMs has also been explored. This method uses self-initiated monomers for MIM synthesis, eliminating the need for external initiators. This approach offers advantages such as high polymerization yields even at high monomer dilutions. In developing CRPP methods, the initiator employed can be substituted with various compounds. For instance, in the nitroxide-mediated precipitation polymerization (NMPP) method, initiators such as AIBN can be replaced with nitroxide compounds. Similarly, in the ILRPP method, the initiator can be replaced by an iniferter agent such as benzyl dithiocarbamate (BDC) [129,199,200].

### 4.4. Temperature

Temperature can play a role in the molecular recognition, selectivity, and adsorption properties of microsphere imprinted polymers. Some research shows that a polymer prepared at 40 °C has the highest enantioselectivity, but that does not necessarily mean the polymer prepared at a lower temperature of 10 °C is inferior. This suggests that temperature can affect microsphere imprinted polymers’ molecular recognition and selectivity. MIMs have good thermal and chemical stability under high or low pH and temperature. This indicates that temperature variations may not significantly impact the stability and performance of microsphere imprinted polymers. Increased temperature facilitated the adsorption of organic pollutants onto MIMs, suggesting that temperature can influence the adsorption capacity of microsphere imprinted polymers [44,91].

At room temperature, stability is achieved when performing non-covalent molecular imprinting and polymerizing heat-sensitive monomers. A higher level of polymerization can be achieved without monomer boiling, making it susceptible to monomer droplets adhering to each other and forming a cream in the solution [129,153,199,201].

### 4.5. Stirring Speed

Stirring speed has some effect on MIMs. Stirring speed plays a role in MIM particle size, conversion, and imprinting efficiency. The stirring speed during the polymerization process affects the particle size of MIMs. Higher stirring speeds tend to result in smaller particle sizes. The impact of stirring speed on the conversion and time to particle formation in MIM synthesis has been investigated. The results suggest higher stirring speeds can achieve the same conversion with increasing reaction time. The molar ratio of the template, monomer, and crosslinker in MIP synthesis can also be influenced by stirring speed. This ratio affects the imprinting efficiency and the formation of specific cavities in the MIM film [202,203].

Furthermore, excessive stirring causes surface shearing and particle fragmentation, while insufficient stirring leads to the separation of the liquid and organic phases, hindering the overall process from homogenizing. This will affect the polymer’s porosity [153,201].

### 4.6. Particle Size

Particle size plays a role in microsphere imprinted polymers’ synthesis, morphology, and performance. The polymer microsphere is limited to a minimum size of 5 nm and a molecular weight of 10,000 Da. This suggests that the particle size of the microspheres used in the imprinting process should be within a certain range. Previous studies have demonstrated that imprinted microspheres and nanoparticles can be synthesized using a simple precipitation polymerization method. Controlling particle size is important to ensure the desired properties and performance of the MIPs. Polymer microspheres possessed micron-sized spherical morphology, spherical particles with a size of 50 microns or approximately 5–2000 μm, adjustable particle size, and porosity. This indicates that the particle size of the microspheres can be adjusted and optimized for specific applications [44,153,201].

### 4.7. pH

The pH of a solution significantly impacts the absorption of target analytes in the SPE process. According to Alipour et al., pH can be utilized as a selective absorbent for MIM nanobeads. In highly acidic and basic media, the extraction efficiency of a compound such as rosmarinic acid is reduced. At low pH values, the active sites of MIM can become protonated, preventing the necessary attractive interactions for the quantitative adsorption of rosmarinic acid onto the MIM absorbent. Consequently, the polymer’s affinity for rosmarinic acid decreases. Additionally, at high pH levels, the adsorption of anionic rosmarinic acid is limited, whereas maximum extraction of rosmarinic acid is observed under neutral conditions (pH approximately 5–7) [125].

## 5. Conclusions

Based on the review conducted, it is evident that MIMs derived from natural products hold significant potential across various applications. Utilizing natural ingredients as the foundational components of MIMs presents opportunities for further exploration in the development of this technology. Despite numerous applications having been identified, there remains untapped potential. Researchers are encouraged to continue exploring advanced methods of MIM fabrication, such as ATRPP, ILRPP, and other techniques. These endeavors can pave the way for new discoveries, enhancing the efficiency and sustainability of MIM technology. The possibility that MIMs could emerge as a primary alternative across various application domains is not discounted. Consequently, further research is anticipated to broaden the understanding and application of MIMs across diverse fields.

## Figures and Tables

**Table 1:** Comparison of MIPs and MIMs.

MIPs	MIMs	Ref.
MIPs are crosslinked polymer networks with specific recognition sites designed to bind to a target molecule.	MIMs are smaller spherical particles with selective binding sites for specific target molecules.	[44,57,93,101,102,103]
They are typically in the form of bulk materials or thin films.	Microspheres are often used in applications where size and shape are necessary, such as drug delivery systems or bioseparation processes.	
	Microspheres have a more uniform size distribution compared to bulk MIPs.	
These polymers are usually synthesized in bulk and ground into small particles for various applications.	These microspheres are synthesized using techniques such as suspension polymerization or emulsion polymerization.	
MIPs can be used in chromatography, sensors, drug delivery, and separation processes.	Microspheres are advantageous in applications where precise control over the size and shape of the imprinted material is crucial.	
The particle distribution in MIPs is often varied, which can lead to clumping and other issues.	Particle distribution in MIMs occurs evenly so that they have a uniform size, which ranges from 0.1 to 100 μm, depending on the MIM method used.	

**Table 2:** The advantages and disadvantages of MIMs.

Advantages	Disadvantages	Ref.
MIMs exhibit consistent particle distribution and size, enabling stable performance, high affinity, and reproducibility. This uniformity also impacts applications such as chromatography and drug delivery.	MIM synthesis can be more complex and requires precise control over polymerization conditions.	[44,91,103,106,107,108,109]
MIMs provide high specificity for target molecules due to more uniform and accessible binding sites, enhancing recognition and effective binding.	MIM production in more advanced methods such as CRRP tends to require higher costs and more sophisticated techniques.	
MIMs are more stable and less prone to degradation over time.	Non-specific interactions may occur due to the uniform structure, potentially interfering with the specific binding of target molecules.	
The surface area of MIMs can be predicted and optimized for specific applications. Controlling this surface area is crucial for reactions and interactions occurring on the polymer surface.	Although MIMs are reusable, their performance may degrade over several cycles, requiring careful regeneration and optimization.	
The uniform microsphere structure allows for better control over the creation and accessibility of binding sites, which is essential for achieving high selectivity and specificity in molecular recognition.	Although uniform MIMs offer better control over binding sites, microspheres may still have limited accessibility for certain target molecules, especially if the binding sites are deeply embedded in the polymer matrix.	

**Table 3:** Separation of active compounds using CRPP.

Natural Product	Sample	Analyte	Monomer; Crosslinker; Initiator; Template	Yield/Purity (Y%/P%) And Adsorption Capacity (AC)	% Recovery	Ref.
Polyphenol	*Emblica officinalis*	Gallic acid	Acrylic acid (AA); EGDMA; AIBN; gallic acid	P: 75–83% AC: N/A	96–98%	[126]
	*Salvia officinalis*	*Salvia Officinalis* Rosmarinic acid	Methacrylic acid (MAA); EGDMA; AIBN; rosmarinic acid	P: N/A AC: N/A	77.80%	[125]

**Table 4:** Separation of active compounds using RAFTPP.

Natural Product	Sample	Analyte	Monomer; Crosslinker; Initiator; Template	Yield/Purity (Y%/P%) And Adsorption Capacity (AC)	% Recovery	Ref.
Aristolochic acids	*Aristolochia manshuriensis*	Aristolochic acid I (AAI)	Acrylic; EGDMA; AIBN; aristolochic acid I	P: N/A AC: 2.72 mg/g	91.50%	[148]

**Table 5:** Separation of active compound using suspension polymerization.

Natural Product	Sample	Analyte	Monomer; Crosslinker; Initiator; Template	Yield/Purity (Y%/P%) And Adsorption Capacity (AC)	% Recovery	Ref.
Alkaloid	*Sophora flavescens* Root	Quinolizidine alkaloids	Acrylamide 1.0 mmol; EGDMA 4.0 mmol; AIBN 0.3 mmol; oxymatrine 0.2 mmol	Y: 80.21–89.15% and 85.33–95.28% AC: 110.8(oxymatrine) and 63.4 mg/g (matrine)	Oxymatrine (80.21–89.15%) and matrine (85.33–95.28%)	[157]
Ginsenoside	American ginseng	Ginsenoside Rb1	Methyl methacrylate and 4-vinylphenyl boronic pinacol ester (4-VBPE); EGDMA; 2,2′-Azobis (2,4-dimethyl) valeronitrile (ABVN); ginsenoside Rb1	Y: N/A AC: 81.45 μmol/g	92.39%	[158]
Flavonoid	*Rhododendron* species	Farrerol, taxifoli, kaempferol, hyperin	4-Vinylpyridine 4.0 mmol; EGDMA 20 mmol; AIBN 0.1 mmol; farrerol 0.1 mmol	Y: N/A AC: 10.04–20.66 mg/g	Farrerol (80.12–105.46%, taxifolin (82.02–95.41%), kaempferol (75.08–89.05%), hyperin (64.04–78.03%)	[159]
Flavonoid	*Larix griffithiana*	Dihydro-quercetin	4-Viniylpyridine 0.065 mmol; EGDMA 0.41 mmol; AIBN 0.15 mmol; dihydroquercetin 0.016 mmol	Y: N/A AC: 77.72 mg/g	74.64–101.80%	[160]
Polyphenol	*Homalomena occulta*, *Cynomorium songaricum*	Protocatechuic acid (PA)	Acrylamide 5.0 mmol; EGDMA 30.0 mmol; AIBN 0.6 mmol; PA 1.0 mmol	Y: 86.3–122% AC: N/A	86.3–102.2%	[40]

**Table 6:** Separation of active compound using precipitation polymerization.

Natural Product	Sample	Analyte	Monomer; Crosslinker; Initiator; Template	Yield/Purity (Y%/P%) And Adsorption Capacity (AC)	% Recovery	Ref.
Polyphenol	*Salvia officinalis*	Rosmarinic acid	Methacrylic acid (MAA); EGDMA; AIBN; rosmarinic acid	P: N/A AC: N/A	77.80%	[125]
Bioflavonoid	*Camellia oleifera*	Proanthocyanidin	Acrylamide; TRIM; AIBN; proanthocyanidin	P: 70.95%, Y: 83.25% AC: 76 mg/g	10.34 ± 2.11% and 85.24 ± 3.05%	[167]
Flavonoid	*Rhododendron* species	Rutin	Methacrylic acid 4.0 mmol; EGDMA 25.0 mmol; AIBN 1.0 mmol; rutin 0.5 mmol	P: N/A AC: 2.43 mg/g	105.98%	[168]
Glycoside	Chinese patent medicines	Rhaponticin	Acrylamide 6.0 mmol; EGDMA 30.0 mmol; AIBN 0.6 mmol; rhaponticin 1.0 mmol	Y: 77.82–91.00% AC: N/A	77.82–91.00%	[169]
Polyphenol	Traditional Chinese Medicine (TCM)	Chlorogenic acid	Methacrylic acid 3.0 mmol; TRIM 5.0 mmol; AIBN 0.15 mmol; chlorogenic acid 0.25 mmol	P: N/A AC: 14.3–23.67 µmol/g	78.85%	[170]

**Table 7:** Separation of active compound using Pickering emulsion polymerization.

Natural Product	Sample	Analyte	Monomer; Crosslinker; Initiator; Template	Yield/Purity (Y%/P%) And Adsorption Capacity (AC)	%Recovery	Ref.
Flavonoid	*Spina gledittsiae*	Quercetin	4-VP; divinylbenzene (DVB); AIBN; quercetin	P: N/A AC: 521 µg/g	N/A	[183]
Steroid	Steroid	17-β-estradiol	MAA; EGDMA; AIBN; 17-β-estradiol	P: N/A AC: N/A	N/A	[184]

## Data Availability

No new data were created or analyzed in this study. Data sharing is not applicable to this article.
